# Delayed sleep onset in depressed young people

**DOI:** 10.1186/1471-244X-14-33

**Published:** 2014-02-08

**Authors:** Nicholas Glozier, Bridianne O’Dea, Patrick D McGorry, Christos Pantelis, Günter Paul Amminger, Daniel F Hermens, Rosemary Purcell, Elizabeth Scott, Ian B Hickie

**Affiliations:** 1 Brain and Mind Research Institute, University of Sydney, Sydney, New South Wales, Australia; 2 Orygen Youth Health Research Centre, Centre for Youth Mental Health, Melbourne, Australia; 3 Melbourne Neuropsychiatry Centre, Department of Psychiatry, University of Melbourne and Melbourne Health, Melbourne, Victoria, Australia; 4 Department of Child and Adolescent Psychiatry, Medical University Vienna, Vienna, Austria

**Keywords:** Depression, Delayed sleep onset, Youth mental health, Clinical staging

## Abstract

**Background:**

The circadian abnormality of delayed sleep phase has been suggested to characterise a subgroup of depressed young adults with different risk factors and course of illness. We aim to assess the prevalence and factors, particularly substance use, associated with such delay in a large help-seeking cohort of young people with mental health problems.

**Methods:**

From a consecutively recruited sample of 802 help-seeking young people, 305 (38%) had at least moderate depressive symptoms (QIDS-C_16_ >10), sleep data and did not have a chronic severe mental illness. Demographic and clinical characteristics were evaluated through self report and clinical interview. Delayed sleep phase was defined as a sleep onset between the hours of 02:00 a.m. – 06:00 a.m. and the characteristics of this group were compared to normal phase sleepers.

**Results:**

Delayed sleep onset was reported amongst 18% (*n =* 56/305) of the depressed group compared to 11% of the non-depressed young people. Amongst the depressed group, delayed sleep onset was associated with tobacco, alcohol and cannabis misuse and short sleep duration (*x̅*: 5.8 hrs *vs. x̅*: 7.8 hrs). There were no differences in demographic factors, personality traits or symptoms. Tobacco smoking was very common: In logistic regression analyses only tobacco use (OR 2.28, 95% CI: 1.04 - 5.01) was associated with delayed sleep onset. There was no interaction with age.

**Conclusions:**

Delayed sleep onset was twice as common in depressed young people as the general population and young people with other mental health problems, and is a potential marker for a subgroup of mood disorders. Those with delayed sleep onset were not more severely depressed but had short sleep duration, a risk for chronic psychological ill health, and higher levels of tobacco use. Nicotine use was common in this group, has biological evidence as a sleep disrupter, and requires specifically addressing in this population.

## Background

Sleep disturbance, usually characterised as ‘insomnia’, is a common occurrence in depression in both primary [1] and secondary [2] care settings, as well as a key feature of the diagnosis. In clinical studies of depressed patients, sleep problems are often the most persistent and/or residual symptoms hampering individuals from remission or recovery [3]. This has led to the development of a diverse range of treatments aimed at improving sleep patterns among those who are depressed [4]. A particular form of sleep disturbance, delayed sleep phase, is often observed in young people with depression: actigraphy measurements have demonstrated that habitual sleep onset later than 1:00 a.m. is seen in 30% of young people with unipolar depression compared to only 10% of age-matched healthy controls [5]. Adolescence and early adulthood is a particularly vulnerable period for the development of delayed sleep-wake cycles. It is hypothesised that a delay in sleep onset typically occurs in adolescence due to changes in melatonin secretion and/or environmental factors which signifies a natural progression for adolescents to fall asleep at a later time when compared to children [6]. However, a clinical pattern, termed Delayed Sleep Phase Disorder (DSPD), may emerge when sleep onset persistently occurs between the hours of 2:00 a.m.–6:00 a.m. and begins to interfere with functioning [7]. DSPD is not always accompanied by a difficulty in falling asleep unless individuals attempt to fall asleep outside the hours of their habitually delayed bedtime. Although DSPD may be accompanied by day time fatigue, delayed sleepers do not consistently report poorer sleep quality [8]. DSPD is relatively uncommon in older adult populations, with onset after 30 years of age rare. However, the estimated point prevalence among young people (12–25 years) falls between 7-10% [9]. Furthermore, while many young people may not meet the full criteria for the disorder, their delayed onset sleeping patterns often represent subclinical forms which may be consistent with a mild diagnosis [10-12]. Young people with delayed sleep onset often demonstrate marked sleep differences on weekends when compared to weekdays [13,14]. This is partly due to the nature of their education and work schedules [15,16] which results in a short weekday sleep duration as the individual limits their sleep to match their sleep chronotype: the degree to which a person organises their daily activities in the morning or evening [17]. Such short sleep duration is a risk factor for the chronicity of psychological distress [18] and mood disorders [19], poorer educational performance [20] and behavioural problems [21,22] whereas the eveningness sleep chronotype is associated with higher levels of depressed mood, conduct problems and hyperactivity [23,24]. This suggests that delayed sleep onset may represent a risk factor, or marker, for a worse trajectory of mental ill health and associated disability.

Some authors have suggested that delayed sleep onset is associated with, and possibly caused by, substance use, particularly tobacco and alcohol [25-27]. It is well known that common psychoactive substances can adversely affect sleep in a dose response manner [28-30] with nicotine a stimulant [31], alcohol both a sedative [32] and stimulant [33], with other drugs having varied effects. With the development of more focused treatment strategies aimed at targeting the different phenotypes and staged trajectories of mood disorders, this study aims to explore the prevalence and patterns of delayed sleep onset among depressed young people presenting to primary mental health care and, in particular, whether there is any association with substance use.

## Methods

### Sample

**Figure 1:**
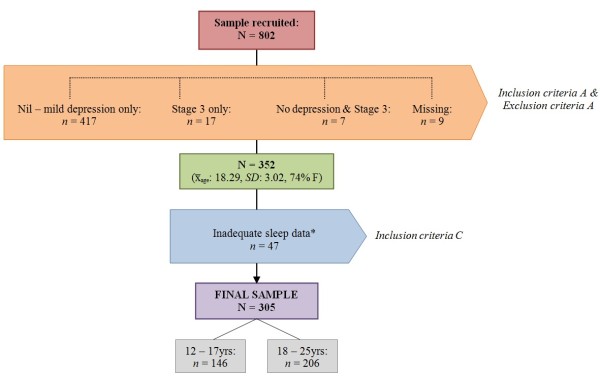
Sample recruitment, inclusion and exclusion criteria.^*^ Excluded participants (*n* = 47) reported significantly lower drive scores on the BIS/BAS measure when compared to final sample (*x̅:* 9.13 *vs. x̅:* 10.26) but no other differences in outcomes measures were found.

Between January 2011 and August 2012, all young people aged between 12–25 years who presented to one of four headspace clinics in Sydney and Melbourne were approached for participation in a longitudinal cohort study evaluating the course of psychiatric disorders among young people, described in full elsewhere [34]. Established by the Australian Government in 2006, Headspace centres provide youth-focused mental health and general health services, drug and alcohol services and vocational assistance to young people aged 12–25 years. There is direct access with no need for a clinician referral and no specific catchment area. There are currently 40 centres located nationally, the four in this study being amongst the first established. The most common reasons for attendance at Headspace are mental health problems, primarily anxiety and depressive symptoms, often in the context of psychosocial issues such as relationship conflict with family and peers [35]. As Headspace focuses on both youth mental health and early intervention, young people may present for care with varying illness severity (e.g. sub threshold symptoms – severe symptoms, mild – severely impaired functioning) across a range of mental health problems [36]. Individuals with a clinician-determined intellectual disability, acute suicidality or those without fluent English were not invited to participate. Eight hundred and two participants were recruited. To limit the sample to those with at least moderate levels of depressive symptoms and sleep data (see Figure 1), but excluding those with a pattern of severe mental illness that has progressed into a distinct psychotic or mood disorder, the following criteria were applied:

#### Inclusion criteria

a) A moderate or greater level of depressive symptoms as determined by the total score on the clinician rated Quick Inventory of Depressive Symptomatology (QIDS-C_16_) [37]. The QIDS-C_16_ assessed the presence, during the previous seven days, of the major DSM-IV diagnostic symptoms of depression, rated on a 4-point Likert scale with total scores ranging from 0–27. Depression severity can then be classified as ‘minimal’ (0–5), ‘mild’ (6–10), ‘moderate’ (11–15), ‘severe’ (16–20) or ‘very severe’ (21–27). The QIDS-C_16_ is considered to be an effective uni-dimensional measure of depression with good reliability (α ≥ 0.8) among youth samples [37].

b) A self-reported habitual sleep onset within ‘normal’ (22:00 – 01:59) or ‘delayed’ hours (02:00 – 06:00) [7,10-12]. As this study focused only on a comparison of normal and delayed phenotypes, participants who reported habitual bedtimes in daylight hours (e.g. 06:01 – 19:59) were excluded (*n* = 8).

#### Exclusion criteria

c) The presence of a recurrent or persistent adult pattern of mental illness or mental disorder that has had incomplete remission and is associated with deteriorating social, educational, occupational functioning due to persistent or reoccurring symptoms. This is described as a Stage 3 mental illness as outlined by the Clinical Staging Model [38].

### Procedure

After the individuals' initial clinical assessment, consenting participants were contacted by a research assistant via telephone or in person to discuss the nature and aims of the research. Participants aged 15 years and over provided written informed consent, while those aged 12–14 years (inclusive) assented with written informed consent provided by a parent or guardian.

Using a structured interview, participants were assessed by research assistants who held graduate degrees in psychology. The structured interview consisted of the clinical measures outlined below in Assessment. Research assistants were trained in the use of the structured interview and achieved an interviewer reliability score of at least 0.8 on each of the interviewer-rated clinical measures before recruitment commenced. The research assistants completed the structured interview with each participant before providing an iPad or laptop for the completion of the self-report measures. This process took approximately 1–2 hours to complete. Participants each received a $20 gift voucher for reimbursement.

### Assessment

#### Sleep

Sleep characteristics were assessed using four adapted items from the Pittsburgh Sleep Quality Index [39] and the first four items of the QIDS-C_16_. *Time spent in bed:* elapsed time between participants’ bed time and wake time. To control for outliers, responses were curtailed to a range of 3 – 15 hours. *Time taken to fall asleep*: minutes taken to fall asleep. *Self-reported sleep duration*: participants’ estimation of how many hours per night they slept. To control for outliers, responses were curtailed to a range of 3 – 15 hours. *Calculated sleep duration*: a validation method in which the time taken to fall asleep was subtracted from the total time spent in bed, for each participant. *Sleep quality:* assessed using a categorical self-rating which was collapsed into two categories: ‘good’ or ‘bad’. *Fatigue:* Using a single question, participants were asked how often they had experienced trouble staying awake in certain activities during the past month which was then coded as ‘persistent’ (three or more times per week) or ‘non-persistent’ (all other responses). Insomnia characteristics were taken from the first four questions of the QIDS-C_16_. *Sleep onset insomnia*: participants who took ‘more than 30 minutes to fall asleep’ for more than half of the time. *Mid-nocturnal insomnia*: participants who reported ‘waking up more than once a night and staying awake for 20 minutes or more’ for more than half of the time. *Early morning insomnia:* participants who reported ‘awakening at least one hour before they needed to’ for more than half the time. *Hypersomnia*: participants who reported sleeping 12 hours or longer in a 24 hour period.

#### Demographics and psychosocial risks

Age, gender, education and employment were obtained through self report [34]. Individuals who did not have a paid position of employment in the past month were categorised as ‘not in employment’. Participants who did not report participating in any type of educational study were categorised as ‘not in education’.

#### Substance use

Participants’ tobacco, alcohol and cannabis use in the past three months was assessed in the clinician interview using the WHO Alcohol, Smoking and Substance Involvement Screening Test (WHO-ASSIST) [40] which also provides a category of risk for each substance. For cannabis and tobacco, scores greater than 3 indicated ‘at risk’ participants and for alcohol, scores greater than 10. ‘At risk’ individuals are at risk of, or already are, experiencing health, social, financial, legal and relationship problems resulting from their substance use. ‘At risk’ also indicates the possibility of dependence.

#### Personality

*Rumination:* was assessed using the short version of the Ruminative Response Scale [41] as rumination has been shown to mediate the association between sleep disturbance and depression [19]. *Behavioural Inhibition& Activation:* The 24-item self-reported Behavioural Inhibition/Behavioural Activation System (BIS/BAS) Scale [42] assessed neuroticism, behavioural inhibition and behavioural activation with subscales for reward responsiveness, drive and fun-seeking.

#### Symptomatology

*Generalised anxiety disorder* and *psychological distress* were assessed using the self-reported Generalised Anxiety Disorder scale (GAD-7) [43] and Kessler’s Psychological Distress scale (K-10) [44]. The presence and severity of *mania* in the previous 48 hours was assessed using the Young Mania Rating Scale (YMRS) [45]; an 11 item interview and behavioural observation. *Suicide ideation* (QIDS-C_16_ item 12) was rated by clinical interview.

#### Functioning

*Social and occupational functioning:* assessed using the clinician-rated Social and Occupational Functioning Assessment Scale (SOFAS) [46] allocating an overall functioning score ranging between 0 – 100, with a higher score suggesting a superior level of functioning. *Disability:* using the 12 item self-report WHODAS12 questionnaire [47], participants were asked to rate their difficulty in daily life activities and participation during the past 30 days. Global scores range from 0 – 100 with higher scores indicating a moderate to severe level of disability. *Quality of life:* a single, self-reported item from the WHOQOL-100 [48].

### Analysis

All statistical analyses were conducted using SPSS Version 21 for Windows. Group differences between delayed and normal sleep onset phenotypes were assessed using *t*-tests or *χ*^2^ tests. Levene’s tests for equal variances were conducted for the continuous clinical variables, to which none violated any assumptions. Response bias amongst those not answering the sleep questions was likewise assessed. Due to the number of *t*-tests conducted for the continuous clinical measures (*n* = 12), a Bonferonni correction was made for the univariate analyses. The adjusted alpha level for statistical significance for reporting such univariate associations was determined to be *p* < .004. However, the multivariate analysis conservatively included all variables with *p* < .05. To account for any potential age effects, all analyses were repeated in a sensitivity analysis, stratifying the sample into two groups (12–17 years *vs*. 18–25 years). To explore the contributing factors for delayed sleep onset, a logistic regression was conducted with the sleep onset phenotype (delayed *vs*. normal) entered as the dichotomous dependent variable. Depression was not entered into the regression model as it was used to define the sample and the measure had demonstrated colinearity with the dependent variable, sleep onset (i.e. items 1–4 of the QIDS-C_16_ assesses sleep). A hierarchical method of logistic regression analysis was employed and only models with non-significant Hosmer-Lemeshow goodness-of-fit tests were included in the results. A final sensitivity analysis using a more restricted exposure, repeated the above approach but categorised those with a sleep onset of 21:00 – 01:59 as ‘normal’ and those from 02:00 – 05:00 as ‘delayed’ onset.

### Ethics

Ethics was granted by the Human Research Ethics Committees at the University of Melbourne and the University of Sydney.

## Results

As outlined in Figure 1, the study sample consisted of 305 participants of the 802 consecutively recruited help-seeking young people aged between 12 – 25 years (*x̅*_age_: 18.36, *SD*: 3.01, 75% female) of whom 64% (*n* = 194/305) reported moderate depression, 29% (*n* = 89/305) severe and 7% (*n* = 22/305) very severe depression (as defined by QIDS-C_16_ cut-offs). The average depression severity score was 14.93 (*SD*: 3.23, range 11 – 26) with no significant gender difference. In this sample, 34% (*n* = 104/305) were in some form of employment, 66% (*n* = 201/305) were in some form of education, with 21% (*n* = 65/305) not participating in either. Tobacco was the substance most frequently used on a daily basis with 36% (*n =* 110/305) of the sample identifying as daily smokers: 30% (*n =* 33/110) of these daily smokers were aged under 18 years. Amongst this sample, 6% (*n =* 18/305) reported daily alcohol consumption and 10% (*n =* 30/305) reported daily cannabis use. A total of 50% (*n* = 152/305) presented ‘at risk’ for tobacco misuse, 19% (*n* = 57/305) for alcohol and 31% (*n* = 93/305) for cannabis. There were no significant differences between gender and substance risk. Sleep disturbances were common: 72% (*n* = 219/305) reported sleep-onset insomnia, 33% (*n* = 100/305) experienced mid-nocturnal insomnia, 23% (*n* = 69/305) early-morning insomnia and 15% (*n* = 46/305) hypersomnia. A total of 70% (*n* = 213/305) reported poor sleep quality and 19% (*n* = 59/305) reported persistent fatigue.

Delayed sleep onset was reported among 18% (*n* = 56/305, *Mdn*_bedtime_ 03:00, *Mdn*_waketime_ 09:30) of the primary sample. Normal sleep onset was reported by 82% (*n* = 249/305, *Mdn*_bedtime_ 22:00, *Mdn*_waketime_ 07:00). For comparison among the non-depressed young people (e.g. QIDS-C_16_ range 0 – 10 and Clinical Stage < 2) in the overall help-seeking sample, the prevalence of delayed sleep was 11% (*n =* 42/417). Outlined in Table 1, a higher level of all types of substance misuse risk was seen in the delayed onset group. There were no significant differences in symptomatology, personality factors, disability or quality of life. Upon going to bed, delayed sleepers did not report taking longer to fall asleep than the normal phenotype; however, the delayed group reported shorter sleep duration, spending two hours less in bed (*x̅:* 6.71 *vs. x̅:* 8.75) and one hour less asleep (*x̅:* 5.89 *vs. x̅:* 6.89). The delayed onset sleepers did not report poorer sleep quality (75%, *n* = 42/56 *vs*. 69%, *n* = 171/249) or more persistent fatigue (16%, *n* = 9/56 *vs*. 20%, *n* = 50/249) than the normal sleep phenotype. There was no interaction of age group with these associations, however, in those aged between 12 – 17 years, alcohol (OR: 2.61, 95% CI: 0 .73 - 9.54) and cannabis misuse (OR: 2.07, 95% CI: 0.72 - 5.92) did not have statistically significant associations with delayed sleep onset due to fewer substance users, but had very similar effect sizes to the overall group.

**Table 1:** Characteristics of normal and delayed sleep onset phenotypes in depressed young people (N = 305)

	**Delayed *n =*** **56**		**Normal *n =*** **249**			
	**Frequency**	**%**	**Frequency**	**%**	**OR (95% CI)**	***p***
*Gender*						.19
Males	18	32%	59	24%	1.5 (0.8 - 2.9)	
Females	38	68%	190	76%		
*Employment*						.98
Not in employment	37	66%	164	66%	1.0 (0.1 - 1.8)	
In employment	19	34%	85	34%		
*Education*						.01
Not in education	27	48%	77	31%	2.1 (1.2 - 3.8)	
In education	29	52%	172	69%		
*High Substance Risk*						
Tobacco	39	71%	113	45%	**2.9 (1.6 - 5.5)**	**.001**
Alcohol	18	33%	39	16%	**2.6 (1.4 - 5.1)**	**.003**
Cannabis	24	43%	69	28%	2.0 (1.1 - 3.6)	.03
*Sleep quality*						
Poor sleep quality	42	75%	171	69%	1.4 (0.7 - 2.7)	.35
Good sleep quality	14	25%	78	31%		
*Fatigue*						
Persistent fatigue	9	16%	50	20%	1.3 (0.6 - 2.9)	.49
Non-persistent fatigue	47	84%	199	80%		
	*x̅*	SD	*x̅*	SD	MD (95% CI)	
Age	18.5	2.3	18.3	3.1	0.2 (-0.5 - 0.9)	.60
Hours spent in bed	6.7	2.7	8.8	2.1	**-2.0 (-2.8--1.3)**	**.000**
Self-reported sleep duration (hrs)	5.9	2.3	6.9	2.1	**-1.0 (-1.6--0.4)**	**.002**
Calculated sleep duration (hrs)	5.8	2.7	7.8	2.3	**-2.0 (-2.8--1.2)**	**.000**
Minutes taken to fall asleep	85.2	68.0	68.9	56.9	16.3 (-0.9 - 33.6)	.06
Fun-seeking	8.1	2.8	9.0	2.7	-0.9 (-1.7 - -0.1)	.03
Drive	10.2	3.2	10.3	2.7	-0.1 (-0.9 - 0.7)	.80
Reward Responsiveness	9.8	3.0	9.6	2.8	0.2 (-0.6 - 1.0)	.65
Inhibition	12.0	4.8	11.4	3.8	0.7 (-0.5 - 1.8)	.24
Rumination	33.3	6.5	32.1	5.5	1.2 (-0.4 - 2.9)	.15
Depression	15.3	3.6	14.9	3.2	0.4 (-0.6 - 1.3)	.42
Anxiety	13.9	5.2	13.0	5.0	0.9 (-0.6 - 2.3)	.24
Mania	5.1	5.7	4.6	5.2	0.5 (-1.0 - 2.1)	.50
Psychological Distress	36.2	7.7	34.7	7.2	1.5 (-0.6 - 3.6)	.16
Suicidal Ideation	9.1	9.2	9.7	8.8	-0.6 (-3.2 - 2.0)	.64
SOFAS score	58.7	10.0	62.3	10.0	-3.6 (-6.5 - -0.7)	.02
WHODAS score	19.9	9.6	17.6	8.7	2.2 (-0.4 - 4.9)	.09
Quality of life	2.5	1.1	2.6	.9	-0.1 (-0.4 - 0.1)	.31

**Bold = adjusted alpha level of** ***p < .004***. SOFAS: Social and Occupational Functioning Assessment Scale, WHODAS: World Health Organisation Disability Assessment Schedule.

Table 2 displays the logistic regression analysis evaluating the factors associated with delayed sleep onset. Age and fun-seeking behaviour were entered as continuous covariates; gender, education, and substance risk were dichotomous covariates. Step 1 confirmed that age and gender were not significantly associated with delayed sleep onset. The observed association of no education status and lower fun-seeking with delayed sleep phenotype was subsequently attenuated to non-significant after adjustment for substance misuse. In step 3, tobacco misuse risk emerged as the only factor significantly associated with delayed sleep onset (adjusted OR: 2.28, 95% CI: 1.04 - 5.01). The sensitivity analysis using the more restricted exposure categories of ‘normal’ (21:00 – 01:59) and ‘delayed’ (02:00 – 05:00) showed no differences in results.

**Table 2:** **Multivariate associations of demographic and clinical factors with delayed sleep onset phenotype in depressed young people (*n*** **= 302)**

	**(i) Base Model C-S *R***^**2.**^**= .004**		**(ii) Adding education & personality C-S *R***^**2**^ **= .033**		**(iii) Adding Substance Use C-S *R***^**2**^ **= .066**	
	**OR**	**95% CI**	**OR**	**95% CI**	**OR**	**95% CI**
Age	1.0	0.9 - 1.1	0.9	0.9 - 1.1	0.9	0.8 - 1.1
Gender	1.5	0.8 - 2.8	1.3	0.7 - 2.5	1.3	0.7 - 2.6
Not in education			**2.0**	1.0 - 3.8	1.9	0.9 - 3.6
Fun-seeking			**0.9**	0.8 - 1.0	0.9	0.8 - 1.1
Cannabis Risk					1.0	.05 - 2.0
Alcohol Risk					1.8	0.9 - 3.8
Tobacco Risk					**2.3**	1.0 - 5.0

***Bold*** *= p < .05. Step 1: Nag R*^*2*^ *= .01, -2LL: 285.30, Model χ*^*2*^*(2) = 1.35, p = .51. Step 2:* Δχ^2^ = 4.33, *Nag R*^*2*^ *= .05, -2LL: 276.41, Model χ*^*2*^*(4) = 10.25, p = .04. Step 3:* Δχ^2^ = 4.24, *Nag R*^*2*^ *= .11, -2LL: 266.03, Model χ*^*2*^*(7) = 20.62, p < .01.*

## Discussion

In this sample of help-seeking depressed young people, habitual delayed sleep onset was common: Nearly one in five participants with depression reported going to bed after 2:00 a.m. As the prevalence of delayed sleep onset in the general population of young people is less than 10%, the same proportion seen in the non-depressed help-seeking young people, these results suggest rates may be higher in young people experiencing moderate – severe depression Although the current participants were not clinically assessed for, or diagnosed with, DSPD, their sleeping patterns align with elements of the current diagnostic criteria [8]. In this sample, delayed sleep onset appeared to result in an average sleep duration of less than six hours and two hours less in bed. Despite this, the delayed sleepers did not report significantly poorer sleep quality or more persistent fatigue. This is consistent with the profile of DSPD found in previous studies of young people [11,16,49]. Given that short sleep duration of less than six hours is associated with the chronicity of mental disorders in young people [18], and a range of poor health outcomes, the delayed onset group may represent a particularly high risk group of depressed young people, though follow-up is needed. Delayed sleep onset accompanied by short sleep duration is consistent with the Clinical Staging Model for mood disorders and has been suggested to signify a circadian pathophysiological profile of depression that may have a different prognosis and response to treatment [4,38,50]. In our studies of young people in the early stages of mood disorders, circadian disruption seems common and a potential avenue for targeted treatments.

In this sample, delayed sleepers did not have significantly different symptom profiles, and, importantly, no greater levels of depression symptom severity. This suggests that delayed sleep isn’t a marker for depression severity in a depressed sample but rather something qualitatively different about this group. Neither did they differ in age, self-reported functioning, employment and disability. Suggested differences in education and function did not withstand adjustment for multiple testing.

In this help-seeking group, delayed sleepers were significantly more likely to report alcohol, and tobacco misuse, with the latter emerging as the strongest, and only independent predictor of delayed sleep onset. Although this might suggest a predisposition to a hedonic or delinquent lifestyle among young people with delayed sleep [51], no associated personality traits were found which undermines this line of thought. Instead, this association is consistent with other authors’ observations that people who smoke are at greater risk of experiencing difficulties falling asleep and mental health problems [52]. There is some biological plausibility too in that nicotine stimulates the cholinergic neurons of the basal forebrain component of the arousal system [53] which are inhibited during both REM and non-REM sleep. Nicotine injections also increase awakenings in rodents [54]. In observed conditions using polysomnography, smokers exhibit an extended sleep latency [55,56] and spectral EEG changes reflecting this arousal [31]. Although cross-sectional, these results suggest that tobacco smoking is associated with delayed and short sleep in depressed youth, which can be helpful when considering treatment interventions. Furthermore, the prevalence of daily tobacco use (38%) in this group is exceptionally high when compared to the national prevalence of 16% [57], particularly for those under 18 where the national prevalence is 4%. This finding is consistent with other help-seeking samples of young people [58] and suggest that this group have an elevated risk of poor health.

### Limitations

Firstly, young people in the early stages of mental illness who present to care often do so in times of crisis, in which they present with a mixture of symptoms. At this early stage, the determination of a primary diagnosis based on conventional criteria, such as depression, may be somewhat unreliable using cross-sectional symptomatology. The high proportion of depressed females in this sample is not necessarily consistent with population prevalence [59] but is consistent with help-seeking youth. Scott et al. [36] found that males presenting to headspace services were more likely to have a primary diagnosis of behavioural or developmental disorders, whereas females were more likely to present with depressive symptoms. Secondly, this study utilised a cross-sectional measurement of sleep in which participants may have reported recent sleep as opposed to habitual [60,61]. As groups were classified based on sleep onset, it may be possible that different associations will emerge using other time points or sleep measures. The addition of objective sleep measures such as actigraphy, or, less practically, polysomnography would significantly improve the reliability and validity of the sleep data. Future studies would also benefit from the use of a chronotype questionnaire, such as the Morningness-Eveningness Questionnaire [62] or Munich Chronotype Questionnaire [63]. Although the levels of depression experienced by this group may have altered their perceptions of sleep, there were no differences in depression (or other symptom scores) between the delayed and normal onset sleep groups which undermines any suggestion of response bias. However, the absence of a non help-seeking control group means that no comparisons can be made with those with no psychiatric disorder. When the substance use analyses were stratified by age, the number of high risk users of alcohol and cannabis in the younger group were substantially smaller in sample size, leading to a similar effect size of association becoming statistically non-significant. Although substance use risk was confirmed by clinical interview, there is a general consensus that some forms of substance use, such as alcohol, may be under-reported, particularly in those under 18 years [58]. As such, the non-significant finding between alcohol and cannabis for those aged less than 18 years should be interpreted with some caution.

## Conclusions

Given that delayed sleep onset and circadian disruption are associated with short sleep duration and other factors indicative of poor prognosis and potentially a vulnerability to a more severe illness [64,65] strategies targeting this disruption warrant testing. As reviewed in Hickie et al. [4] currently these include a range of behavioural, phototherapeutic and melatonin based interventions. The results here suggest another reason to address smoking which may be contributing to poor sleep and may also form part of a circadian treatment approach. With simple non-invasive assessments such as actigraphy and smart phone applications readily available to help identify such a delayed sleep phase in clinical practice, the broader adaptation of more personalised treatments is possible. Determining the efficacy, practicality and acceptability of such adjunctive or stepped approaches alongside current psychopharmacological and psychotherapeutic approaches to treating depression in young people should the next focus.

## Competing interests

Professor Hickie is a Board Member of Psychosis Australia Trust. From 2012, he has been a Commissioner in Australia’s new National Mental Health Commission. He was until January 2012 a director of headspace: the national youth mental health foundation. Prof Hickie was previously chief executive officer (till 2003) and clinical adviser (till 2006) of beyondblue, an Australian National Depression Initiative. He is supported principally for clinical research in depression and health services and population health initiatives related to anxiety and depression by an NHMRC Australian Medical Research Fellowship (2007–12) and now by an NHMRC Senior Principal Research Fellowship (2013–17). He has led projects for health professionals and the community supported by governmental, community agency and pharmaceutical industry partners (Wyeth, Eli Lily, Servier, Pfizer, AstraZeneca) for the identification and management of depression and anxiety. He has received honoraria for presentations of his own work at educational seminars supported by a number of nongovernment organisations and the pharmaceutical industry (including Pfizer, Servier and Astra Zeneca). He has served on advisory boards convened by the pharmaceutical industry in relation to specific antidepressants, including nefazodone, duloxetine, and desvenlafaxine. He leads an investigator-initiated study of the effects of agomelatine on circadian parameters (supported in part by Servier but also by other NHMRC funding) and has participated in a multicentre clinical trial of agomelatine effects on sleep architecture in depression and a Servier-supported study of major depression and sleep disturbance in primary care settings. In addition to national and international Government-based grant bodies, investigator-initiated mental health research at the BMRI he has been supported by various pharmaceutical manufacturers (including Servier and Pfizer) and not-for-profit entities (including the Heart Foundation, beyondblue and the BUPA Foundation). Dr Daniel Hermens is currently supported by a grant from the NSW Ministry of Health, Mental Health and Drug & Alcohol Office as well as an NHMRC Australia Fellowship (awarded to Professor Hickie). He has received honoraria for educational seminars from Janssen-Cilag and Eli Lilly. Dr Elizabeth Scott is the (unpaid) Clinical Director of Headspace Services at the BMRI, the (unpaid) Co-ordinator of the Youth Mental Health Research Program at the BMRI, and Deputy Director of St Vincent’s Private Hospital Young Adult Mental Health Unit. She has received honoraria for educational seminars related to the clinical management of depressive disorders supported by Servier and Eli-Lilly pharmaceuticals. She has participated in a national advisory board for the antidepressant compound Pristiq, manufactured by Pfizer.

## Authors’ contributions

The study was designed and conducted by PM, NG, IH, RP, CP, PA, DH, E.S. The analysis of data and interpretation was conducted by BOD and NG. The paper was prepared by NG, BOD and critically revised by PM, IH, CP, PA, DH, RP, E.S. All authors read and approved the final manuscript.

## Pre-publication history

The pre-publication history for this paper can be accessed here:

http://www.biomedcentral.com/1471-244X/14/33/prepub
